# Pollen Release Dynamics and Daily Patterns of Pollen-Collecting Activity of Honeybee *Apis mellifera* and Bumblebee *Bombus lantschouensis* in Solar Greenhouse

**DOI:** 10.3390/insects10070216

**Published:** 2019-07-22

**Authors:** Hong Zhang, Zhiyong Zhou, Jiandong An

**Affiliations:** 1 Key Laboratory for Insect-Pollinator Biology of the Ministry of Agriculture and Rural Affairs, Institute of Apicultural Research, Chinese Academy of Agricultural Sciences, Beijing 100093, China; 2 State Key Laboratory for Biology of Plant Diseases and Insect Pests, Institute of Plant Protection, Chinese Academy of Agricultural Sciences, Beijing 100193, China

**Keywords:** pollen availability, pollinators, foraging behaviour, pollination, microclimate in greenhouse

## Abstract

Pollen is important not only for pollination and fertilization of plants, but also for colony development of bee pollinators. Anther dehiscence determines the available pollen that can be collected by foragers. In China, honeybees and bumblebees are widely used as pollinators in solar greenhouse agriculture. To better understand the effect of solar greenhouse microclimates on pollen release and pollen-foraging behaviour, we observed the anther dehiscence dynamics and daily pollen-collecting activity of *Apis mellifera* and *Bombus lantschouensis* during peach anthesis in a solar greenhouse in Beijing. Microclimate factors had a significant effect on anther dehiscence and bee foraging behaviour. The proportion of dehisced anthers increased with increasing temperature and decreasing relative humidity and peaked from 11:00 h to 14:00 h, coinciding with the peak pollen-collecting activity of bees. On sunny days, most pollen grains were collected by the two pollinators within two hours after anther dehiscence, at which time the viability of pollen had not yet significantly decreased. Our study helps us to better understand the relationship between food resources and pollinator foraging behaviour and to make better use of bees for pollination in Chinese solar greenhouses.

## 1. Introduction

Bees are important pollinators for many flowering plants. Bees visit flowers to collect nectar and pollen to use as food. Pollen provides protein, lipids, vitamins and minerals for foragers and is considered the most essential nutrient source of adult bees and bee larvae [1,2]. The pollen-foraging behaviour of bees not only affects their colony reproductive success but also the fertilization success of flowers they visit [3].

The pollen-foraging behaviour of social bees is affected by many factors. Studies have shown that the brood and pollen storage in honeybee colony have an important influence on pollen-foraging behaviours of workers [4,5,6,7]. For example, pollen-foraging behaviours increase when pollen is removed or when brood increases in the honeybee colony [5]. Weather conditions also affect the foraging activity of bees, both directly and indirectly. On the one hand, bee foraging behaviour usually occurs within a suitable range of air temperature and humidity [8,9,10]. On the other hand, weather conditions affect bee foraging behaviour by altering the quantity and quality of food resources [11]. Bees carry out foraging behaviour according to the amount of food resources provided by the flowers they visit [12]. When there are insufficient floral resources, bees show a low frequency of foraging trips, even in the presence of ideal abiotic factors [13]. Pollen availability might be a significant factor in determining which flower a bee visits [14,15]. Bumblebees are able to assess pollen content in open flowers visually and make foraging decisions accordingly [16,17]. Anther dehiscence determines the pollen that can be collected by pollinators [18,19]. Consequently, anther dehiscence dynamics should play an important role in the daily pattern of bee pollen-collecting activity, especially in greenhouses, where floral resources are limited.

In recent years, protected cultivation of vegetables and fruits has been rapidly developing for the highly profitable inter-seasonal harvest in China [20]. The most popular cultivation system in northern China is solar-powered plastic greenhouse [21]. Unlike conventional greenhouses where heating and cooling are usually provided by fossil fuel or electric heaters, this type of cultivation system uses only solar energy for crop production. Both honeybees and bumblebees have been used for the pollination of fruit and vegetables in Chinese solar greenhouses [22]. However, the effects of microclimatic conditions in solar greenhouses on pollen release and bee pollen-foraging activity have not been well studied or are confined to only a few Chinese studies.

The aims of the present study were to determine pollen release dynamics throughout the anthesis of peach *Prunus persica* “Okubo” and to record the daily pollen-collecting activity of the honeybee *Apis mellifera* and the bumblebee *Bombus lantschouensis* in solar greenhouse. We discuss the pollen-foraging behaviour of bees in relation to microclimate and pollen availability under solar greenhouse conditions. We seek to better understand the relationship between food resources and pollinator foraging behaviour and to make better use of bees for pollination in Chinese solar greenhouses.

## 2. Material and Methods

### 2.1. Study Organisms

**Figure 1:**
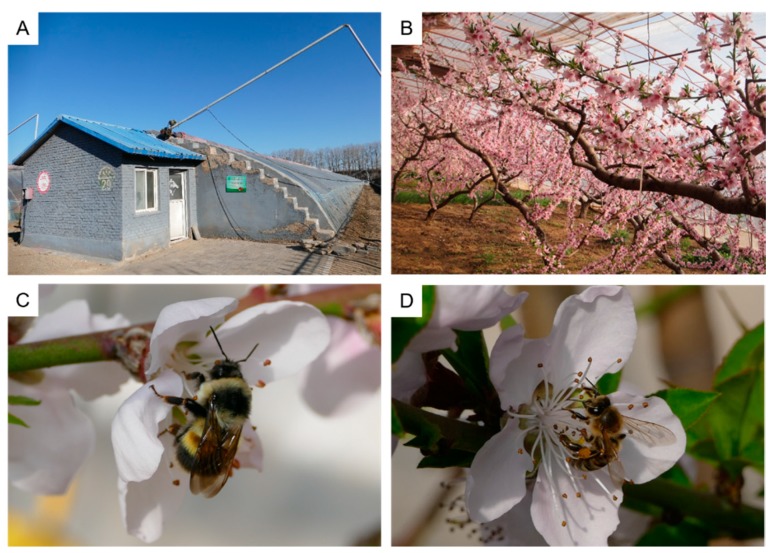
Peach solar greenhouse and bee pollinators. (**A**) Solar greenhouse in northern China; (**B**) peach trees (*Prunus persica* “Okubo”) in solar greenhouse; (**C**) worker of *Bombus lantschouensis* visiting peach flower; and (**D**) worker of *Apis mellifera* visiting peach flower.

This study was carried out in a peach solar greenhouse orchard located in Beijing, China. Like most greenhouses in northern China, the greenhouses in the orchard used solar energy as the only energy source. All the greenhouses were managed in a uniform mode by experienced fruit growers and were controlled at the same temperature and relative humidity (RH) condition. The sunny side of the greenhouse was covered with two polyethylene film layers that overlapped each other on the top of the greenhouse, and one of the layers can be opened and closed (Figure 1A). During the day, the temperature and RH were regulated by adjusting the opening range of the layers. The peach greenhouses were equipped with fly netting on the top to prevent bees from flying out. In each solar greenhouse, 85–90 peach trees (*Prunus persica* “Okubo”) were planted in rows spaced 3 m apart, with 1.5 m spaces within the rows (Figure 1B). The experiments were conducted in the peach blooming period, from 26 January to 5 March in 2014 and from 1 February to 9 March in 2015. One of the greenhouses in the orchard was selected and was split into two sections in the middle by fly net for separate pollination by honeybees and bumblebees (Figure 1C,D). At the beginning of peach flower blooming period, an *A. mellifera* colony, consisting of three frames of approximately a total of 6000 workers, and a *B. lantschouensis* colony, consisting of approximately 60 workers, were placed in each section. Both honeybee and bumblebee colonies were provided by the Institute of Apicultural Research, Chinese Academy of Agricultural Sciences.

### 2.2. Peach Anther Dehiscence

The anther dehiscence observation experiments were conducted on sunny days during blooming in 2015. To observe anther dehiscence dynamics in the greenhouse, newly opened flowers were marked at one hour intervals, from 9:00 h to 14:00 h. During the peach blooming period, 30 newly opened flowers were marked at 9:00, 10:00, and 11:00 h, respectively. And 60 newly opened flowers were marked at 12:00, 13:00, and 14:00 h, respectively. Anther dehiscence of each marked flower was observed and recorded hourly. The temperature and RH were recorded hourly by a mini data logger (174H, Testo, Lenzkirch, Germany).

Besides the observation experiments in the greenhouse, the dehiscence dynamics of anthers on detached filaments and detached whole flowers were analysed in a 5 × 3 split block design in lab. Detached filaments and flowers were collected randomly from 5–8 trees in the greenhouse where we observe peach anther dehiscence dynamics. The collection method of detached filaments and flowers followed Gradziel [23]. Specifically, undamaged anthers were detached at the base of filaments from newly opened flowers, and normal-appearing flowers were collected at pre-blooming stage. Detached filaments and flowers were immediately placed in Eppendorf tubes with moist paper (1.5 mL tubes for anthers and 50 mL tubes for flowers) and were equilibrated for 10 hours at 10 °C to similar hydration and temperature states [24]. Detached filaments and whole flowers were exposed to a range of temperatures (15 °C, 20 °C, 25 °C, 30 °C and 35 °C) and RH conditions (15%, 33% and 64%). Higher RH was established with saturated salt solutions (33% with MgCl_2_, 64% with NaNO_3_), and a lower RH of 15% was established with solid CaSO_4_ and pure water [25]. Anther dehiscence was recorded hourly within a 10-h period. For each experimental unit, at least three filament groups (each group contained 10 undamaged filaments) and at least six undamaged flowers were observed. Filaments/flowers were positioned on a Petri dish that was mounted above saturated salt solutions/pure water in desiccators. An auto recorder was placed in each desiccator to monitor temperature and RH (174H, Testo, Lenzkirch, Germany). 

### 2.3. Pollen-Collecting Activity of Bees

The pollen-collecting activity of bees was observed on sunny days during the full-bloom stage of the peach trees. The behaviour data were obtained from a total of six days in 2014 and five days in 2015. Returning foragers at the hive entrance were monitored by video monitoring (HB7100, Hanbang Technology, Beijing, China) at half hour intervals, for a duration of 10 min, from 9:30 h to 16:00 h. Pollen foragers were determined based on the presence or absence of pollen in the corbiculae. Given that the colony size of the honeybees was much larger than that of bumblebees, we used the proportion of pollen foragers to compare the pollen-collecting behaviour of honeybees and bumblebees. The number of pollen foragers and the total returning foragers at the hive entrance were recorded. The number of pollen foragers at the hive entrance divided by the number of total returning foragers was calculated as the proportion of pollen foragers. To analyse the influence of microclimate on bee-foraging behaviours, the temperature, RH and light intensity were recorded within the first 10 minutes of each half hour from 9:30 h to 16:00 h. Temperature and RH were recorded by auto recorder (174H, Testo, Lenzkirch, Germany). Light intensity was measured by a portable luxmeter (HD 2302.0, Delta OHM, Caselle di Selvazzano, Italy).

### 2.4. Pollen Removal by Bees 

We compared the pollen removal by honeybees and bumblebees during the peak period of anther dehiscence in the peach greenhouses. Pollen removal was measured by counting the residual pollen grains from anthers on flowers visited by honeybees/bumblebees. In the morning of sunny days, peach flowers at pre-bloom stage were selected randomly and isolated individually in fabric mesh bags (2 mm). At 11:00 h, newly opened flowers in bags were selected and marked (We chose to mark newly opened flowers at 11:00 h because the peak period of anther dehiscence was from 11:00 h to 14:00 h based on our observations). Some of these newly opened flowers were uncovered and exposed to honeybees/bumblebees, and some flowers were still bagged as a control. All the marked flowers were observed hourly and dehisced anthers were marked lightly on the filament with a marker pen. Anthers were collected hourly in the first three hours and then at 24 h after anther dehiscence. Anthers from bagged flowers that were not visited by bees were also collected as controls. Each anther was cut off at the top of the filament with sharp scissors and was stored separately in a clean Eppendorf tube. In each treatment (flowers visited by honeybees, flowers visited by bumblebees, bagged flowers), 10 anthers from three to four flowers were collected at each time point. The pollen count method used was adapted from Zhang’s method [22]. Specifically, anthers in Eppdendorf tubes were dried in oven at 55 °C for three hours to make sure that anther dehisced completely. Then anthers were stained with malachite green solution (25 μL 0.1% malachite green aqueous solution dissolved in 10 mL 1% NaCl aqueous solution) for 15 h. An ultrasonic bath was used to separate pollen grains from anthers (JY92-Ⅱ DN, Ningbo, China). The pollen grains were collected by vacuum filtration, using a filter membrane with a pore size of 20 μm. Then the filter membrane was placed on a slide and images were captured by a scanner (Nikon Coolscan 9000 ED, Toyko, Japan). Image J 1.48 was used to count pollen grains in each image (National Institute of Health, Bethesda, MD, USA).

### 2.5. Viability of Pollen in Flowers and That Carried by Bees

The TTC (2,3,5-triphenyl tetrazolium chloride) test was used to determine pollen viability [26]. The pollen viability was detected at one hour, two hours and three hours after anther dehiscence. To avoid any visitors’ influence on pollen viability, flowers at pre-bloom stage were selected randomly and bagged individually in the morning. Newly opened flowers were selected and marked at 11:00 h. To collect anthers at different time after dehiscence, marked flowers were observed at 10 min intervals. Newly dehisced anthers were marked lightly on the filament with a marker pen. At each hour after dehiscence, anthers were collected using sharp scissors, and each anther was stored separately in a clean Eppendorf tube. Pollen from newly dehisced anthers was also tested as a control treatment. In each treatment (one hour, two hours and three hours after anther dehiscence and newly dehisced anthers), approximately 50 anthers from three to five flowers in the greenhouse were collected and tested. All anthers were sampled on sunny days. A viability test was conducted within 20 min after the anther was detached from the filament. Freshly collected anther was sowed at 1% TTC solution (1 g TTC dissolved in 100 mL 0.1 M phosphate-buffered saline, pH = 7.4) and stirred by pipette tip gently to make sure that pollen grains dispersed well in TTC solution. About 10 μL of TTC solution contained with pollen grains was added onto a microscope slide and the slide was covered with a coverslip immediately. Slides were incubated at 35 °C for 15 min. When pollen grains were inculated with TTC solution, viable pollen grains showed colour reaction in dark pink or light pink and dead pollen grains showed no colour reaction. A stereomicroscope (SZX 16, Olympus, Tokyo, Japan) with a CCD camera (DP 7, Olympus, Tokyo, Japan) was used to collect images of sample slides. The viable pollen grains and dead pollen grains were scored in each sample slide. The number of viable pollen grains divided by the number of total pollen grains was calculated as the viability of pollen.

The viability of the pollen carried by pollen foragers was tested. Pollen foragers that returned to the hives were caught individually at the hive entrance every half an hour from 9:30 h to 15:00 h. For honeybee and bumblebee, pollen grains clinging to the body, instead of the pollen grains in the wet corbicular pollen loads, were more valuable for plant fertilization success. So, we measured the viability of body pollen. We extracted the pollen grains clinging to body of honeybees and bumblebees using the method presented by Vaissière et al. [27]. Specifically, bees were anesthetized with CO_2_. The hind legs with pollen were removed using scissors. Each bee was placed in a 2 mL microcentrifuge tube that contained 1.5 mL phosphate-buffered saline and was vortexed for 60 s. The bee body was removed, and the pollen was obtained by centrifugation and was tested by the TTC method. In total, approximately 10–20 honeybees and 6–14 bumblebees were caught for the pollen viability test at each time point. To reduce worker losses in bumblebee colony caused by sampling, three bumblebee colonies of the same colony size and status were brought into the greenhouse in series. In each colony, 30–40 bumblebee workers were sampled. For the removal of pollen foragers might result in a shift of foraging behaviours in bumblebee colony, the sampling of pollen foragers were conducted after all the other behaviour observations ended. After the sampling work, we put new colony in the greenhouse for pollination.

### 2.6. Data Analysis

All statistical analyses were performed using IBM SPSS 20 (Chicago, IL, USA). For anther dehiscence, we tested how temperature and RH affected anther dehiscence. A general linear repeated measure model was used with the proportion of dehiscent anthers on detached whole flowers and the proportion of dehiscent anthers on detached filaments as response factors, time (hourly for the first 10 h for anthers on detached whole flowers and on detached filaments) as the within-subject factor, and temperature (15 °C, 20 °C, 25 °C, 30 °C and 35 °C) and RH (15%, 33% and 64%) as the between-subject factors based on a binomial distribution. Mauchly’s Test of Sphericity indicated that the assumption of sphericity was violated (*p* < 0.001); therefore, a Greenhouse–Geisser correction was used (ε = 0.75). 

For the bee pollen-collecting activity, we investigated whether the proportion of pollen foragers differed between bee species and/or over time. The behaviour data in 2014 and 2015 was combined together for no difference existed in two years’ data (*F*_1,304_ = 0.243, *p* = 0.622). A general linear repeated measure model was used with pollen-forager proportion as the response variable, time (each half an hour from 9:30 to 16:00 h) as the within-subject factor and bee species (honeybee and bumblebee) as the between-subject factor based on a binomial distribution. Mauchly’s Test of Sphericity indicated that the assumption of sphericity was violated (*p* < 0.001); therefore, a Greenhouse–Geisser correction was used (ε = 0.43). Before independent-sample T tests were used to compare the differences in pollen-collecting behaviour between honeybees and bumblebees at each time point, a Shariro–Wilk normality test was used to test normality and a Levene test was used to test homoscedasticity. Multiple regression was used to determine whether the number of pollen foragers could be predicted based on environmental factors (temperature, RH and light intensity). We used the number of pollen foragers as the predicted variable, and the numbers of honeybees and bumblebees were predicted separately due to the considerable difference in the quantity of individual numbers of the two bee species. Before we built the regression model, we tested whether a linear relationship existed between the dependent variable and each independent variables by drawing scatterplots. The independence of residuals was checked using the Durbin–Watson statistic and the normality of the residuals was tested by a normal Q–Q plot. The homoscedasticity was assessed by plotting the studentized residuals against the unstandardized predicted values. The multicollinearity was detected through VIF values.

We compared the pollen removal by honeybees and bumblebees during the peak period of anther dehiscence in the peach greenhouse. A general linear model was used with pollen residual as the response variable and pollination method (honeybee pollination, bumblebee pollination, restricted pollination), time (one hour, two hours, three hours, and 24 h after anther dehiscence) and their two-way interaction as fixed factors based on a Poisson distribution. Post-hoc pairwise comparisons were used to test the significant differences among the levels of pollination method and time.

For pollen viability dynamics during anther dehiscence, pollen viability data had significantly different variances (Levene’s test, *F*_3,209_ = 4.746, *p* = 0.003); thus, the non-parametric Kruskal–Wallis H test followed by the Dunn–Bonferroni post-hoc method was used to test whether differences existed in the viability of pollen collected at different times since anther dehiscence. Data on the viability of pollen carried by bees also had significantly different variances (Levene’s test, *F*_23,210_ = 2.583, *p* < 0.001), and the non-parametric Mann–Whitney U method was used to test the difference in the viability of pollen carried by different bee species at every time point.

## 3. Results

### 3.1. Peach Anther Dehiscence Dynamics and Influential Factors

**Figure 2:**
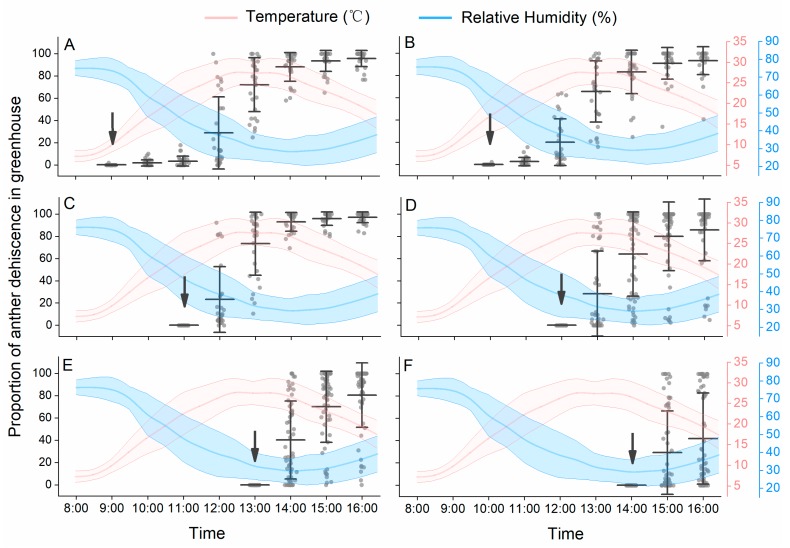
Peach anther dehiscence dynamics under solar greenhouse conditions. The panels (**A**–**F**) represent anther dehiscence dynamics of flowers opened at different time of day. The arrows indicate the time when flowers fully opened. Data are presented as the mean ± S.D.

Under solar greenhouse conditions, the temperature increased from 6 °C to 30 °C, and the RH decreased from 77 to 28% from 8:00 h to 13:00 h. Then, the temperature decreased over time combined with increasing RH in the afternoon (Figure 2). On sunny days, peach flowers bloomed continuously in the daytime. Most peach anthers begin to dehisce after the flowers have bloomed. For flowers that opened in the early morning (from 9:00 h to 10:00 h), few anthers dehisced within the first one to two hours (Figure 2A,B), at which time the temperature was low and the RH was high. The highest proportion of anther dehiscence occurred from 11:00 h to 14:00 h, and most anthers dehisced within two hours after blooming, when the temperature increased to 25 °C and RH dropped to 40% (Figure 2C,D). For flowers that opened after 14:00 h, at which time the temperature began to decrease and RH began to increase, most anthers did not dehisce in the day (Figure 2E,F).

**Figure 3:**
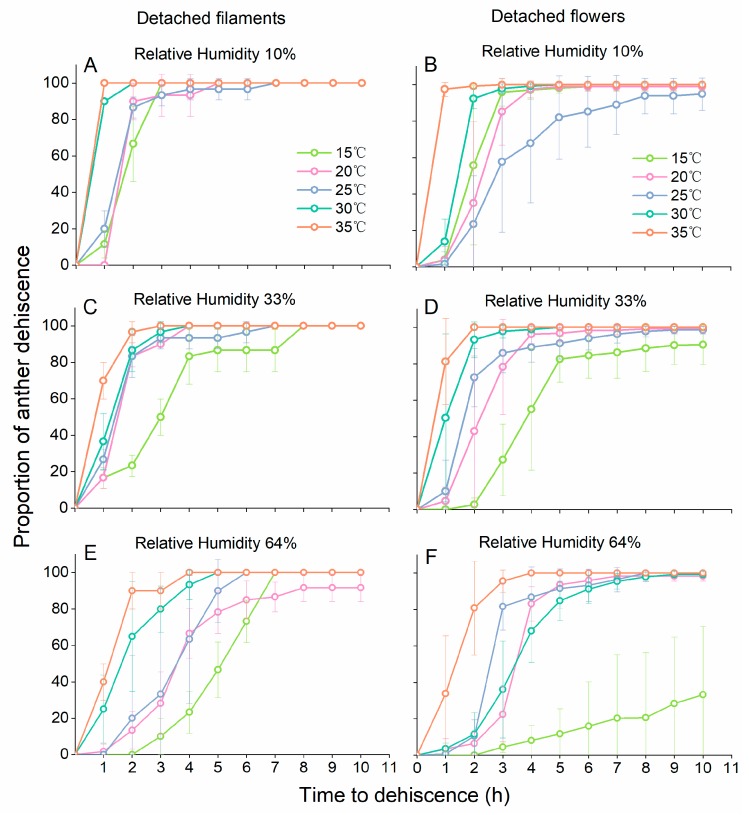
Effect of temperature and relative humidity on time to anther dehiscence in detached filaments and detached flowers. The panels (**A**,**C**,**E**) represent anther dehiscence in detached filaments at 10% RH, 33%RH and 64% RH, and the panels (**B**,**D**,**F**) represent anther dehiscence in detached flowers at 10% RH, 33%RH and 64% RH. Data are presented as the mean ± S.D. At least three filament groups (each group contained 10 undamaged filaments) and at least six undamaged flowers were observed in each experimental unit.

Anther dehiscence was suppressed with increasing RH and decreasing temperature, for both detached filaments and detached flowers (Figure 3). The results of the GLM repeated measures analysis showed that the interaction among temperature, RH and time was significant for anther dehiscence of detached flowers (temperature × RH × time interaction: *F*_23.694, 210.284_ = 7.815, *p* < 0.001). The dehiscence dynamics of anthers on detached filaments were also significantly influenced by temperature and RH conditions (temperature × RH × time interaction: *F*_22.891, 117.317_ = 6.304, *p* < 0.001). The dehiscence dynamics of anthers on detached filaments at different temperatures were similar when RH was low (Figure 3A,C,E). For anthers from whole detached flowers, small changes in temperatures often resulted in larger differences in dehiscence patterns (Figure 3B,D,F). At 64% RH, only a few anthers in detached flowers dehisced at 15 °C (Figure 3F).

### 3.2. Bee Pollen-Collecting Activity in the Peach Greenhouse

**Figure 4:**
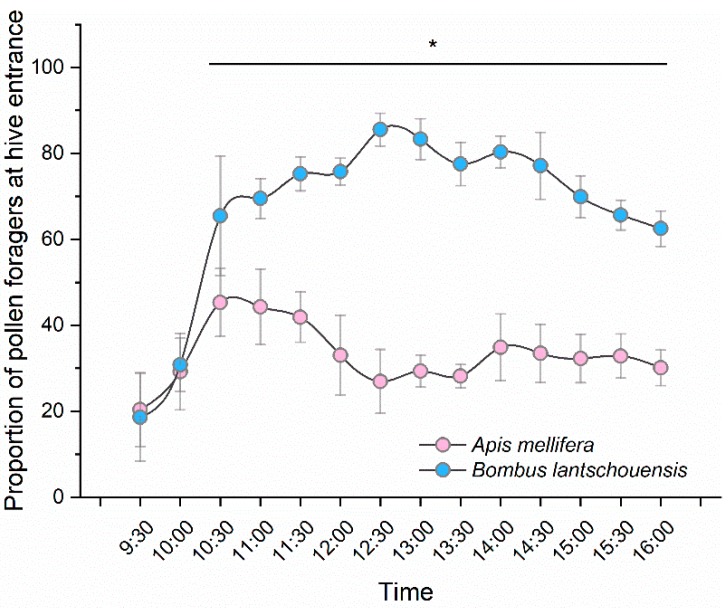
Daily pattern of pollen-collecting activity of *Apis mellifera* and *Bombus lantschouensis* in the peach solar greenhouse. The behaviour data were obtained from eleven days and a total of 154 behaviour records were obtained for both the honeybee colony and bumblebee colony. Data are presented as the mean ± S.D. The “*” indicates significant difference between the proportion of pollen foragers of honeybee and bumblebee. The number of pollen foragers at the hive entrance divided by the number of total returning foragers was calculated as the proportion of pollen foragers. Independent-sample T tests were used to compare the differences in pollen forager proportions between honeybees and bumblebees at each time point (Table S1).

The daily pattern of pollen-collecting activity was different between the two species (bee species × time interaction: *F*_5.520,110.394_= 51.234, *p* < 0.001, Table S1). Because of the larger colony size, the number of honeybee pollen foragers was much higher than that of bumblebees. When considering the proportion of pollen foragers relative to total foragers, bumblebees showed a greater preference for collecting pollen than honeybees (*F*_1,20_ = 662.794, *p* < 0.001). The proportions of bumblebee pollen foragers were significantly higher than those of honeybees at each time point (*p* < 0.001), except for 9:30 h (*p* = 0.667) and 10:00 h (*p* = 0.629). For bumblebees, the number of pollen foragers at the hive entrance increased with time in the morning and reached a peak at the middle of the day. Honeybees usually preferred to collect pollen in the morning, and the pollen-collecting activity reached its peak between 10:30 h and 11:30 h, whereas fewer honeybees collected pollen in the afternoon (Figure 4).

The pollen-collecting activity of bees in the greenhouse was influenced by weather conditions. Multiple regression analysis was performed to predict the number of pollen foragers from light intensity, temperature and RH. Multicollinearity was not observed in either model, with all VIFs less than 2.5. All of these variables significantly predicted the number of honeybee and bumblebee pollen foragers (Table 1). For example, the pollen-collecting activity of honeybees was most strongly predicted by temperature (β = 0.495), followed by light intensity (β = 0.347). 

### 3.3. Pollen Removal by Bees

**Figure 5:**
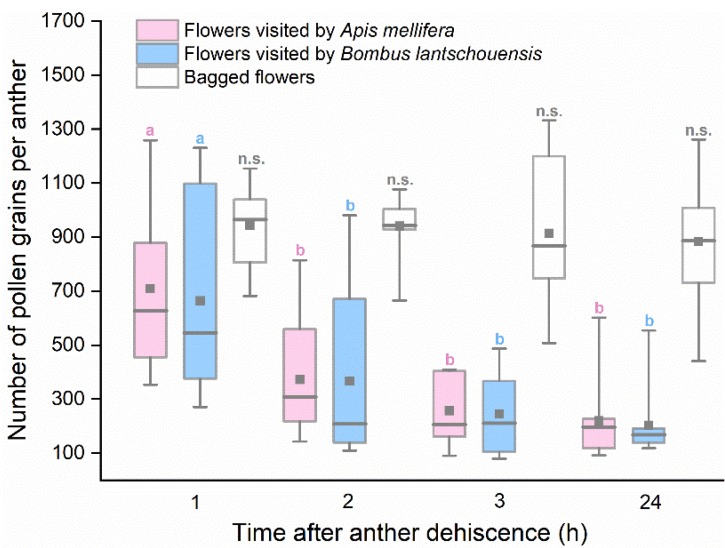
Number of residual pollen grains in anthers from flowers visited by *Apis mellifera* and *Bombus lantschouensis.* Different letters indicate significant differences in number of pollen grains among different time based on the Duncan test at α = 0.05. Boxes indicate quartiles with the median marked as a horizontal line and the mean marked as a dot.

The general linear model revealed a significant interaction between pollination method and time on pollen removal (pollination method × time interaction: *F*_6,119_ = 2.460, *p* = 0.029). For bagged flowers, no significant difference existed in residual pollen quantity since anther dehiscence (*F*_3,39_ = 0.181, *p* = 0.909). However, the residual pollen quantity of anthers exposed to bees differed significantly over time (*F*_3,79_ = 17.155, *p* < 0.001). The number of residual pollen grains decreased rapidly within the first two hours and remained steady after three hours of exposure to bees (Figure 5). There was no significant difference in the pollen quantity decline dynamics of flowers visited by different bees (*F*_1,79_ = 0.138, *p* = 0.711).

### 3.4. Viability of Pollen in Flowers and that Carried by Bees

**Figure 6:**
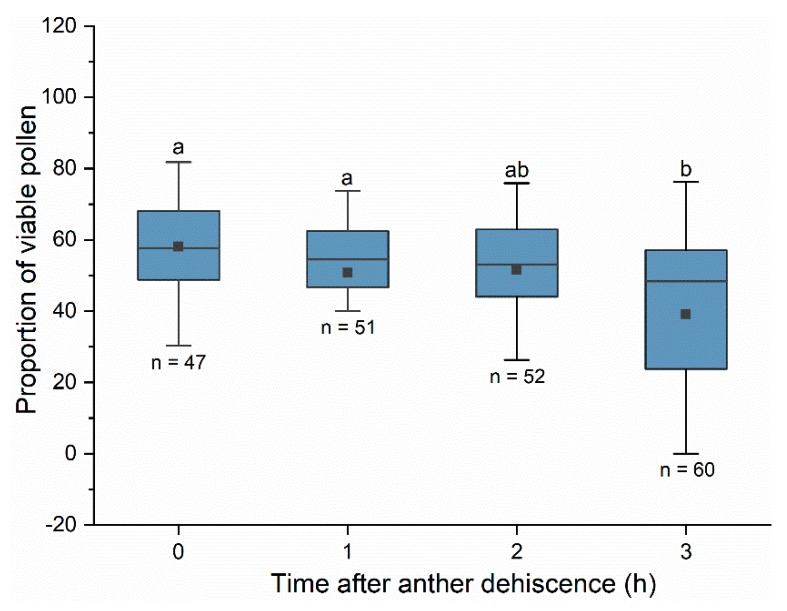
The proportion of viable pollen at different times after anther dehiscence. Pollen viability was tested using TTC. The difference in pollen viability at different times was compared using the Kruskal–Wallis test. Different letters indicate significant differences in pollen viability based on the Dunn–Bonferroni test at α = 0.05. Boxes indicate quartiles with the median marked as a horizontal line and the mean marked as a dot.

Under solar greenhouse conditions, peach flower pollen viability decreased significantly after anther dehiscence (Figure 6, Kruskal–Wallis test: H = 16.685, df = 3, *p* < 0.001). The proportion of viable pollen was approximately 58% at the time of anther dehiscence, and the viability declined to 50% one to two hours later. Approximately three hours later, the proportion of viable pollen was less than 50%. A post-hoc test revealed that the proportion of viable pollen three hours after anther dehiscence was significantly lower than that at the time of dehiscence (t = 3.936, *p* < 0.001) and one hour (t = 2.753, *p* = 0.006) after anther dehiscence. There was no significant difference in the proportion of viable pollen within two hours of anther dehiscence (*p* = 0.235).

**Figure 7:**
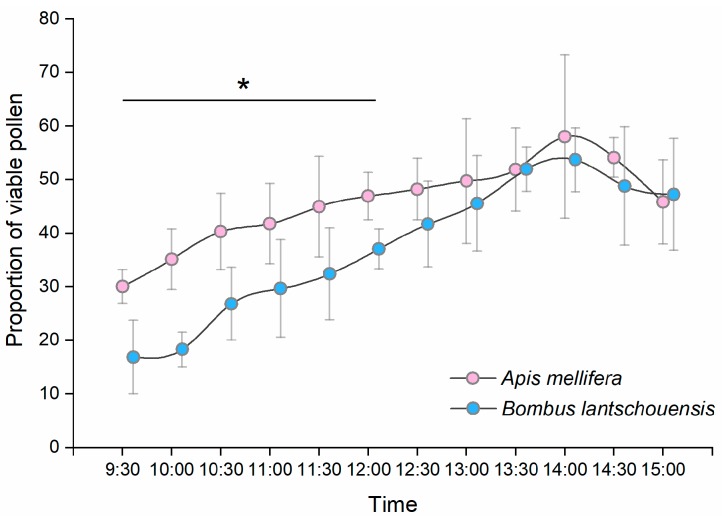
Temporal differences in the proportion of viable pollen carried by *Apis mellifera* and *Bombus lantschouensis* in the peach solar greenhouse. Data are presented as the mean ± S.D. The difference in the viability of pollen carried by *Apis mellifera* and *Bombus lantschouensis* was tested by the Mann–Whitney U method. The “*” indicates significant differences (*p* < 0.05) between *A. mellifera* and *B. lantschouensis* at each time point.

The proportion of viable pollen carried by honeybees and bumblebees differed significantly over time during the day (Figure 7). The proportion of viable pollen carried by both bee species increased over time in the morning and reached a peak at approximately 14:00 h and then decreased in the afternoon. From 9:30 h to 12:00 h, the pollen carried by honeybees had higher viability than that carried by bumblebees, and there was no significant difference in the viability of pollen carried by different bee species after 12:30 h (Table S2).

## 4. Discussion

Anther dehiscence has been shown to be sensitive to abiotic stress in the environment [25,28,29,30]. Most of the anthers dehisced normally under solar greenhouse conditions in our study. Anther dehiscence is induced by certain combinations of environmental factors, of which temperature and RH are the two most important [28]. Gradziel and Weinbaum assessed the influence of RH on anther dehiscence of apricot, peach and almond and found that anther dehiscence was suppressed under increasing RH [24]. We found that both low temperature and high RH suppressed the dehiscence of anthers on detached filaments and detached flowers. In our study, anther dehiscence dynamics showed strong dependence on the diurnal change of temperature and RH. Although the daily variation of temperature and RH was high based on our measurements, most anthers dehisced normally under such conditions on sunny days. Few anthers in freshly opened flowers dehisced before 10:00 h, and the peak dehiscence time was usually from 11:00 h to 14:00 h, with rising temperature and decreasing RH. For flowers that bloomed after 14:00 h in the afternoon, at which time the temperature was below 25 °C and the RH was higher than 40%, most anthers did not dehisce until the next day.

The peak daily pollen-collecting activity of bees occurred when high numbers of anthers dehisced in our study. Pollen availability in the environment has an important influence on bee pollen-foraging behaviours. Sabugosa-Madeira et al found a significant positive correlation between total airborne pollen and weight of pollen collected by honeybees [31]. In our study, few bees carried pollen to the hive entrance before 10:00 h. The proportion of pollen foragers increased significantly after 10:30 h, when more anthers began to dehisce and bees were able to collect more pollen. Differences were found in the daily changes in the proportions of honeybee and bumblebee pollen foragers. The proportion of bumblebee pollen foragers increased consistently until the middle of the day and then declined gradually in the afternoon. During our experiment, we found that many young honeybees conducted orientation flights at approximately 12:30–14:00 h, especially in sunny weather, which resulted a lower proportion of pollen foragers in honeybee colony during the middle of the day. In honeybee colonies, when bees are approximately one week old, they take orientation flights in front of the hive and nearby [32]. When using a video monitoring system to record the behaviours of bees coming in and out of the hive entrance, it was difficult to distinguish foragers without pollen and bees taking an orientation flight, which resulted in a high number of ‘total foragers’. Another explanation for the lower proportion of honeybee pollen foragers was that more honeybees visited flowers for nectar. However, in our experiments, we did not observe nectar-collecting behaviour of bees. Further studies are needed to compare the pollen- and nectar-foraging behaviours of honeybees and bumblebees in solar greenhouses.

In our study, the bee pollen-collecting behaviours were influenced by environmental conditions in the solar greenhouse. Many studies have reported that weather conditions affect the foraging behaviours of bees [9,13,33]. Compared with RH, temperature and light intensity are more important weather conditions that affect honeybee foraging flights [10]. We found similar results in our study. Although the RH significantly predicted the number of pollen foragers in the regression model, RH was less influential than temperature and light intensity within the range that occurred in our solar greenhouse. Bees can perceive light intensity changes [34,35] and use it as a cue in foraging tasks [36]. In our study, both bee species became active with increasing light intensity during daytime in the greenhouse. Of the two bee species, the pollen-collecting activity of *A. mellifera* was more highly associated with temperature than that of *B. lantschouensis.* It was reported that the active foraging temperature is 9–10 °C for bumblebees and 15–16 °C for honeybees [37,38]. Bumblebees are known to perform better than honeybees when the temperature is low, in both the greenhouse and open field [39]. It is possible that the lowest temperature recorded in our study did not challenge the cold tolerance of bumblebees. 

During the peak period of anther dehiscence in the day, both honeybees and bumblebees were able to collect viable pollen. Pollen has the highest viability at the time of anther dehiscence. In our study, the proportion of viable pollen decreased significantly three hours after anther dehiscence. By counting the residual pollen in anthers from flowers exposed to bees, we found that the quantity of pollen decreased rapidly within two hours after anther dehiscence, which means that bees collected most of the pollen before the viability decline. The high efficiency of viable pollen transfer makes a huge contribution to the fertilization success of plants. By sampling pollen foragers at different times of the day, we found that the proportion of viable pollen carried by bees increased over time in the morning and reached a peak at approximately 13:30–14:30 h. Given the anther dehiscence dynamics, we assumed that in the early morning, most pollen collected by bees was from anthers that dehisced the previous day, with a relatively lower viability. In the middle of the day, most anthers dehisced within two hours after flower blooming, and bees had a greater opportunity to collect pollen with higher viability.

In our study, the viability of pollen carried by bees was much lower than the newly released pollen. In some pollinator-plant systems, pollen viability is significantly reduced when it comes into contact with the body of insects [40]. Pollen viability may also decrease as a result of pollinator grooming behaviour [41,42]. Differences were also found in the proportion of viable pollen carried by honeybees and bumblebees. In the early morning, pollen carried by honeybees had a much higher viability than pollen carried by bumblebees. Some studies have compared the viability of pollen carried by different pollinators. Compared with flies and ants, Apidae generally carry more viable pollen [43,44]. We assumed that the different preferences of honeybees and bumblebees for flowers could explain the results. In our previous study, we found that honeybees specialized in a higher frequency of visits to flowers at the peak stage, when the pollen viability was high, whereas bumblebees visited different flowers more randomly, resulting in higher viability of pollen carried by honeybees [45]. However, in our study, we did not consider the differences in grooming behaviour and flight duration between honeybees and bumblebees. Whether those differences resulted in the difference in pollen viability between the bee species remains to be tested. Due to the labour intensive nature of sampling work, we only measured the viability of pollen carried by bees in only one greenhouse and we didn’t examine variations among different colonies. As forager behaviour can differ greatly between colonies, much more research needs to be done to obtain more reliable results. 

## 5. Conclusions

In this study, we investigated peach anther dehiscence dynamics and the pollen-collecting activities of *A. mellifera* and *B. lantschouensis* in solar greenhouses. The microclimate in the solar greenhouses ensured the normal pollen release of peach flowers and bee pollen-foraging behaviours. The peak of anther dehiscence dynamics coincided with the peak pollen-collecting activity of bees in the solar greenhouses. Both honeybees and bumblebees adapted well to the microclimate in the solar greenhouses and were highly efficient in collecting viable pollen during the peak period of anther dehiscence. When using managed bees for pollination in greenhouses, more attention should be paid to the temperature and RH control, which is not only crucial to pollen release, but also important for bees’ pollination performance. 

## Figures and Tables

**Table 1:** Effect of light intensity, temperature and RH on pollen-collecting activity of *Apis mellifera* and *Bombus lantschouensis*. The model to predict honeybee pollen-collecting activity was as follows: number of pollen foragers = 0.718 light intensity (×10^−3^ lux) + 2.317 temperature (°C) − 0.420 RH (%) − 43.311. The model to predict bumblebee pollen-collecting activity was as follows: number of pollen foragers = 0.363 light intensity (×10^−3^ lux) + 0.323 temperature (°C) − 0.068 RH (%).

Parameters	Number of Pollen Foragers in *A. mellifera*	Number of Pollen Foragers in *B. lantschouensis*
**Light intensity (×10^−3^ Lux)**		
Unstandardized Coefficients (B)	0.718	0.363
Std. Error	0.162	0.037
Standardized Coefficients (β)	0.347	0.591
t	4.432	9.920
*p*	0.000	0.000
**Temperature (°C)**		
Unstandardized Coefficients (B)	2.317	0.323
Std. Error	0.426	0.096
Standardized Coefficients (β)	0.495	0.233
t	5.442	3.364
*p*	0.000	0.001
**RH (%)**		
Unstandardized Coefficients (B)	−0.420	−0.068
Std. Error	0.099	0.022
Standardized Coefficients (β)	−0.309	−0.170
t	4.268	−3.080
*p*	0.000	0.002
**Constant**		
Unstandardized Coefficients (B)	−43.311	−3.554
Std. Error	10.131	2.286
t	−4.275	−1.555
*p*	0.000	0.122
Adjusted R^2^	0.457	0.684
*F*	43.879	111.599
*p*	0.000	0.000
Num. behaviour records	154	154

## Supplementary Materials

The following are available online at https://www.mdpi.com/2075-4450/10/7/216/s1, Table S1: The difference in the pattern of daily pollen-collecting activity between Apis mellifera and Bombus lantschouensis, Table S2: Comparison of the viability of pollen carried by Apis mellifera and Bombus lantschouensis.
