# Supplementary material for: Pollen Release Dynamics and Daily Patterns of Pollen-Collecting Activity of Honeybee Apis mellifera and Bumblebee Bombus lantschouensis in Solar Greenhouse
Source: Insects. 2019 Jul 22;10(7):216. doi: 10.3390/insects10070216 (PMC6681390; doi:10.3390/insects10070216)
Supplement: Supplementary file 1 [file insects-10-00216-s001.zip › Table S/Table S1.docx]

| Species |  | Time | | | | | | | | | | | | | | Sum | | *F* | *p* |
| --- | --- | --- | --- | --- | --- | --- | --- | --- | --- | --- | --- | --- | --- | --- | --- | --- | --- | --- | --- |
|  |  | 9:30 | 10:00 | 10:30 | 11:00 | 11:30 | 12:00 | 12:30 | 13:00 | 13:30 | 14:00 | 14:30 | 15:00 | 15:30 | 16:00 |  |  |  |  |
| *A. mellifera* | Mean | 20.5 | 29.2 | 45.3 | 44.3 | 41.9 | 33.0 | 26.9 | 29.3 | 28.1 | 34.9 | 33.5 | 32.3 | 32.8 | 30.1 | 33.0 | 12.173 | | 0.000 |
|  | S.D | 8.5 | 8.9 | 7.9 | 8.8 | 5.8 | 9.3 | 7.4 | 3.7 | 2.7 | 7.7 | 6.8 | 5.6 | 5.1 | 4.1 | 9.4 |  |  |  |
| *B. lantschouensis* | Mean | 18.6 | 30.8 | 65.4 | 69.5 | 75.2 | 75.7 | 85.6 | 83.3 | 77.6 | 80.3 | 77.1 | 70.0 | 65.7 | 62.5 | 66.9 | 120.998 | | 0.000 |
|  | S.D | 10.2 | 6.2 | 13.9 | 4.6 | 4.0 | 3.1 | 3.8 | 4.8 | 5.0 | 3.7 | 7.8 | 4.9 | 3.5 | 4.1 | 19.6 |  |  |  |
| Sum | Mean | 19.54 | 30.0 | 55.4 | 56.9 | 58.6 | 54.4 | 56.2 | 56.3 | 52.9 | 57.6 | 55.3 | 51.1 | 49.3 | 46.3 | 50.0* | 76.108* | | 0.000* |
|  | S.D | 9.2 | 7.5 | 15.1 | 14.6 | 17.7 | 22.9 | 30.5 | 27.9 | 25.6 | 24.0 | 23.5 | 19.9 | 17.3 | 17.1 | 22.9* |  |  |  |
| *t* |  | -0.436 | 0.490 | 4.176 | 8.416 | 15.677 | 14.437 | 23.251 | 29.713 | 28.772 | 17.581 | 14.015 | 16.736 | 17.620 | 18.437 | 662.794* | | (*F*=47.033, *p*=0.000)# | |
| *p* |  | 0.667 | 0.629 | 0.000 | 0.000 | 0.000 | 0.000 | 0.000 | 0.000 | 0.000 | 0.000 | 0.000 | 0.000 | 0.000 | 0.000 | 0.000* | |  |  |

**Table S1 The difference in the pattern of daily pollen-collecting activity between *Apis mellifera* and *Bombus lantschouensis***

* *F* statistic and *p* value of the main effect; # *F* statistic and *p* value of the interaction effect.
