# Supplementary material for: Pollen Release Dynamics and Daily Patterns of Pollen-Collecting Activity of Honeybee Apis mellifera and Bumblebee Bombus lantschouensis in Solar Greenhouse
Source: Insects. 2019 Jul 22;10(7):216. doi: 10.3390/insects10070216 (PMC6681390; doi:10.3390/insects10070216)
Supplement: Supplementary file 1 [file insects-10-00216-s001.zip › Table S/Table S2.docx]

**Table S2 Comparison of the viability of pollen carried by *Apis mellifera* and *Bombus lantschouensi****s*

|  | Time | | | | | | | | | | | |
| --- | --- | --- | --- | --- | --- | --- | --- | --- | --- | --- | --- | --- |
|  | 9:30 | 10:00 | 10:30 | 11:00 | 11:30 | 12:00 | 12:30 | 13:00 | 13:30 | 14:00 | 14:30 | 15:00 |
| U | 92 | 130 | 313 | 72 | 80 | 75 | 28 | 61 | 68 | 38 | 35 | 14 |
| Z | 3.356 | 4.001 | 4.343 | 2.433 | 2.430 | 3.067 | 1.521 | 0.836 | 0.495 | 0.578 | 1.356 | -0.560 |
| *p* | <0.001 | <0.001 | <0.001 | 0.012 | 0.012 | <0.001 | 0.132 | 0.412 | 0.628 | 0.574 | 0.181 | 0.589 |
